# Bioremediation of heavy metals using an endophytic bacterium *Paenibacillus* sp. RM isolated from the roots of *Tridax procumbens*

**DOI:** 10.1007/s13205-016-0560-1

**Published:** 2016-11-12

**Authors:** M. Govarthanan, R. Mythili, T. Selvankumar, S. Kamala-Kannan, A. Rajasekar, Young-Cheol Chang

**Affiliations:** 1 Department of Applied Sciences, College of Environmental Technology, Muroran Institute of Technology, 27-1, Mizumoto, Muroran, Hokkaido 050-8585 Japan; 2 PG and Research Department of Biotechnology, Mahendra Arts and Science College (Autonomous), Kalippatti, Namakkal, 637501 Tamil Nadu India; 3 Division of Biotechnology, Advanced Institute of Environment and Bioscience, College of Environmental and Bioresource Sciences, Chonbuk National University, Iksan, 54596 South Korea; 4 Environmental Molecular Microbiology Research Laboratory, Department of Biotechnology, Thiruvalluvar University, Serkadu, Vellore, 632115 Tamil Nadu India

**Keywords:** Bioremediation, Endophytes, Heavy metals, *Tridax procumbens*

## Abstract

The aim of the present study was to assess the bioremediation potential of endophytic bacteria isolated from roots of *Tridax procumbens* plant. Five bacterial endophytes were isolated and subsequently tested for minimal inhibitory concentration (MIC) against different heavy metals. Amongst the five isolates, strain RM exhibited the highest resistance to copper (750 mg/l), followed by zinc (500 mg/l), lead (450 mg/l), and arsenic (400 mg/l). Phylogenetic analysis of the 16S rDNA sequence suggested that strain RM was a member of genus *Paneibacillus*. Strain RM also had the capacity to produce secondary metabolites, indole acetic acid, siderophores, 1-aminocyclopropane-1-carboxylate (ACC) deaminase, and biosurfactant and solubilize phosphate. The growth kinetics of strain RM was altered slightly in the presence of metal stress. Temperature and pH influenced the metal removal rate. The results suggest that strain RM can survive under the high concentration of heavy metals and has been identified as a potential candidate for application in bioremediation of heavy metals in contaminated environments.

## Introduction

Heavy metal contamination of soil and water has become a serious problem worldwide. Bioconcentration and subsequent biomagnification of heavy metals and high levels of toxicity they impart to biological organisms indicate the necessity for the removal of heavy metals from contaminated soil and water (Govarthanan et al. 2014). Several physical, chemical, and biological methods have been proposed for the removal of heavy metals from contaminated soil and water (Shi et al. 2009), of which bacteria-based bioremediation is considered as a promising and viable method. Bacteria-based bioremediation is less expensive and eco-friendly (Govarthanan et al. 2015a), but is highly influenced by several factors, such as survival of bacteria in contaminated soil or water, influence of abiotic factors on the growth of bacteria, mechanism of metal detoxification, expression rate of metal detoxificiation genes, and influence of pollutants on bacterial activity (Suja et al. 2014; Govarthanan et al. 2015b; Praburaman et al. 2015).

In the past few years, attention has been concentrated on the bioremediation of heavy metals using endophytic plant-growth-promoting bacteria (Sun et al. 2010; Ma et al. 2015). Endophytes or endophytic bacteria colonise internal tissues of normal healthy plants with little negative effects on the host (Schulz and Boyle 2006). The endophytic bacterial strains are ubiquitously distributed in a wide range of plant species, and have been found in plant organs, such as leaves, stem, root, flowers, and fruits (Compant et al. 2011; Sun et al. 2010). These bacteria play a significant role in plant growth and development by producing plant-growth-promoting substances, such as indole-3-acetic acid (IAA), siderophores, and 1-aminocyclopropane-1-carboxylate (ACC) deaminase (Tiwari et al. 2016). They also produce biosurfactants and extracellular polymeric substances that prevent, moderate, or nullify the toxicity of heavy metals in these plants (Rajkumar et al. 2009; Weyens et al. 2010). Zhu et al. (2014) reported that endophytic bacteria isolated from hyperaccumulator plants *Pteris vittata* and *Pteris multifida* showed significant metal removal and plant-growth-promoting properties.

*Tridax procumbens* is a widespread weed with massive root system and rapid growth rate, and is commonly seen throughout the year (Khandare et al. 2011). Kumar et al. (2013) reported that *T. procumbens* effectively removed Cr, Cu, Ni, Pb, and Cd from soil contaminated with industrial wastes. However, the endophytic bacterial population in the roots of *T. procumbens* roots and their heavy metal bioremediation potential has not been reported. Hence, the aim of the present study was (1) to isolate metal-resistant endophytic bacteria from roots of *T. procumbens*, (2) to evaluate multi-metal tolerance and plant-growth-promoting characteristics of the isolates, (3) to assess bioremediation potential of the isolate, and (4) to optimise experimental variables, such as pH and temperature for enhanced bioremediation.

## Materials and methods

### Isolation of heavy metal-resistant endophytic bacteria from Tridax procumbens root

Healthy *T. procumbens* plants were collected randomly from agricultural land, and plant sample was washed with tap water followed by several rinses with sterile water. The washed plant was dissected into roots and stems with a sterile scalpel. Root samples were surface sterilised with 95% ethanol and 3% hypochlorite solution for 2–3 min. Surface sterilised root samples were again washed several times in sterile water to remove the sterilization agents. Sterilised root tissue (1 g fresh weight) was ground in a mortar and pestle with 5 ml saline (0.85% NaCl). The saline suspension was serially diluted and plated using the spread plate technique onto Nutrient Agar (Hi-Media, India) plates. The plates were incubated at 35 ± 2 °C for 48 h and observed for bacterial growth on agar surface. Morphologically distinct colonies were purified and stored at 4 °C for further studies.

### Heavy metal tolerance

Heavy metal tolerance levels of isolates were assessed by agar-dilution method. Briefly, the isolates were inoculated in Luria–Bertani (LB) agar plates containing different concentrations of heavy metals (As, Cu, Zn, and Pb) ranging from 100 to 750 mg/l. The plates were incubated overnight at 25 °C and observed for bacterial growth. Lowest concentration of the metal that completely inhibited the growth of bacteria was considered as minimal inhibitory concentration (MIC). All the metal salts were added to LB agar after autoclaving and cooling to 50 °C from filter-sterilised stock solutions (Kamala-Kannan and Krishnamoorthy 2006).

### Plant-growth-promoting properties of the isolates

Indole acetic acid (IAA) production was analysed according to Gordon and Weber. Freshly grown bacterial culture was inoculated into Dworkin and Foster (DF) minimal medium supplemented with 0.5 mg/ml of tryptophan and incubated at 30 °C for 48 h. After incubation, 1 ml bacterial culture was mixed with 2 ml Salkowski’s reagent (150 ml conc. H_2_SO_4_, 250 ml distilled water, and 7.5 ml 0.5 MFeCl_3_·6H_2_O), and allowed to stand at 37 °C for 20 min. Appearance of a pink colour in the tubes indicated IAA production (Gordon and Weber 1951).

Siderophore production efficiency of the isolates was tested according to Schwyn and Neilands by Chrome Azurol S agar (CAS) method. Briefly, 1.0 ml CAS solution was added to 1.0 ml filtered supernatant of the isolate. The siderophore production was estimated by the colour change from blue to orange. Tubes in CAS solution without the supernatant were used as a control.

The 1-aminocyclopropane-1-carboxylate (ACC) deaminase activity of the isolated strains was determined according to Praburaman et al. (2015). In brief, the isolate was inoculated in DF minimal medium supplemented with 3 mM ACC as the nitrogen source. The tubes were incubated at 37 °C on a rotatory shaker at 200 rpm for 48 h. The development of turbidity in the tubes was considered ACC deaminase positive.

Phosphate solubilization efficiency of the isolates was assessed using Pikovskaya medium (Pikovskaya 1948). Briefly, fresh bacterial culture was inoculated into Pikovskaya agar containing inorganic phosphate and incubated at 30 °C for 48 h. Formation of a clear zone around the bacterial colony was considered as an index of solubilization of mineral phosphate.

Biosurfactant producing ability of the endophytes was identified using oil displacement test and emulsification assay. The emulsification assay was calculated using emulsification index:

$${\text{E24 }}\left( {\text{Emulsification index}} \right) = \frac{\text{Height of emulsification layer developed}}{\text{Total height of liquid medium}} \times 100.$$

### Identification of the potential endophytic bacteria

DNA was extracted from strain RM, using QIAGEN (CA, USA) DNA extraction kit, and its concentration was determined using a UV–Vis spectrophotometer (NanoDrop 2000). Fragments of 16S rDNA were amplified using universal primers 27f (5′-AGAGTTTGATCCTGGCTCAG-3′) and 1492r (5′-CCCCGTCAATTCATTTGAGTTT-3′). PCR product was purified using a QIAGEN PCR purification kit and sequenced using an automated ABI PRISM 3700 sequencer (USA). The obtained 16S rRNA sequence was compared against the available sequence database using BLAST program in NCBI website. Phylogenetic tree was constructed using neighbour-joining distance method by software Mega 6.0.

### Effect of heavy metals on growth kinetics of the isolate

Strain RM was inoculated into 250 ml Erlenmeyer flasks containing 100 ml of LB broth and incubated in a shaking incubator at 200 rpm for 24 h at 37 °C. At the stage of the late exponential phase, 100 mg/l of the metal solutions (Cu, Pb, Zn, and As) were inoculated into the culture flasks. Bacterial growth was determined by measuring the optical density (600 nm) (UV-1800, Shimadzu, Japan) at prescribed time intervals (0, 8, 12, 24, and 48 h). All the experiments were performed in triplicates. Growth of the isolate without metals was considered as the control for this experiment.

### Removal of metals at different pH and temperature conditions

The effect of pH on the removal of heavy metals was determined at various pH levels ranging from 6 to 9. In brief, 10 ml of the bacterial suspension was aseptically inoculated into 250 ml Erlenmeyer flasks containing 100 ml of metal solutions (Cu, Pb, Zn, and As) (initial metal concentration 50 mg/l) individually. The flasks were incubated in a shaking incubator (200 rpm) at 37 °C, and the samples were collected after 48 h of incubation. The samples were centrifuged (10,000 rpm for 5 min), filtered (0.22 μm), and analysed for the residual heavy metal concentration using atomic adsorption spectroscopy (AAS) (Thermo Scientific™ iCE™ 3500). Similarly, the effect of temperature was measured by incubating the flasks at different temperatures (30, 35, and 37 °C). Flask without bacteria was used as a control for this experiment. All the experiments were repeated for three times, and each sample was tested in triplicate.

### Bioremediation of Cu, Pb, Zn, and As by the isolate at optimum pH and temperature

Batch experiments were performed to assess the bioremediation potential of the isolate RM. In brief, strain RM was inoculated in 250 ml Erlenmeyer flasks containing 100 ml of LB broth amended with 50 mg/l of Cu, Pb, Zn, or As individually. The flasks were incubated at 37 °C and agitated at 200 rpm for 48 h. Samples were collected at after 48 h, centrifuged (10, 000 rpm for 5 min), filtered (0.22 μm), and analysed for residual heavy metal concentration using AAS.

## Results and discussion

Bioremediation has been widely accepted as an eco-friendly approach for the removal of metals from contaminated soil and water. In the present study, an endophytic bacteria isolated from *T. procumbens* was assessed for their heavy metal bioremediation potential. Five heavy metal-resistant endophytic bacterial strains were isolated from roots of *T. procumbens*, and the isolates were screened for metal resistance on LB agar plates (1/4 strength) supplemented with Cu, Pb, Zn, or As. All the five endophytes showed resistance to the metals, while strain RM showed the highest MIC (Cu; 750 mg/l) strength to all the studied metals. Minimal inhibitory concentration of the isolates is shown in Table 1. The metal resistance appears to be little lower than that typically reported for other plants (Shin et al. 2012). However, a direct comparison of our results with other studies is difficult, because various biotic and abiotic factors highly influence the bacterial metal detoxification rate and bioavailability of metals to the bacterial system (Kamala-Kannan and Krishnamoorthy 2006).

**Table 1:** Multi-metal resistance pattern of the endophytes isolated from the roots of *Tridax procumbens*

Bacterial isolates	As (mg/l)	Zn (mg/l)	Cu (mg/l)	Pb (mg/l)
RM	400	500	750	450
RM1	200	350	150	100
RM2	200	250	200	100
RM3	150	200	350	300
RM4	200	150	200	250

Endophytic bacteria have the ability to improve growth and remediation of heavy metal contaminated environments by producing IAA and other plant-growth-promoting factors (Zhang et al. 2011). Thus, the isolates were screened for their plant-growth-promoting properties. All the isolates successfully produced IAA from tryptophan (Table 2). Strain RM exhibited maximum IAA production (17.2 mg/l). Recent studies have reported endophytes isolated from the roots of *P. vittata* to produce 18.5 mg/l of IAA (Zhu et al. 2014; Tiwari et al. 2016). Siderophores are high-affinity iron-chelating proteins that play a vital iron in making iron available to the bacterial system. Thus, the isolates were screened for siderophore production, and the results are depicted in Table 2. Among the five isolates, strain RM3 and strain RM produced siderophores. The results are in accordance with previous study that reported variations in siderophore production among endophytic bacteria (Tiwari et al. 2016). The isolates RM1, RM4, and RM produced ACC deaminase (see Table 2), an important enzyme that increases availability of nitrogen sources to the bacterial system by degrading ACC into α-ketobutyrate and ammonia, and also regulates ethylene levels in plant systems. The results are in agreement with previous study reporting ACC deaminase activity of endophytic bacteria in *Phytolacca Americana* (Zhang et al. 2015). Phosphate solubilization potential is very common among endophytic bacteria (Ghosh et al. 2016). In this study, a clear zone around the isolates RM2, RM3, and RM colonies in Pikovskaya medium indicated the phosphate solubilization potential of the isolates. The isolates may solubilise the phosphate either by producing enzymes or by organic acids.

**Table 2:** Plant-growth-promoting characteristics of the isolates

Bacterial isolates	Siderophore production	IAA (mg/l)	ACC	Phosphate solubilization	Biosurfactant production
RM	+	17.2 ± 1.2	+	+	+
RM1	−	10 ± 1	+	−	−
RM2	−	8 ± 1	−	+	−
RM3	+	8.2 ± 2	−	+	−
RM4	−	11.1 ± 3	+	−	−

(+) and (−) symbols indicates the presence or absence of different plant-growth-promoting characteristics in the isolated endophytes

All the isolates were also screened for their biosurfactant production ability. Among the five isolates, strain RM showed biosurfactant producing ability. Biosurfactant production is considered as an attracting characteristic of endophytes for environmental application, because biosurfactants detoxify the pollutants present in the contaminated soil and water (De Franca et al. 2015). Based on its multi-metal resistance mechanisms, plant-growth-promoting characteristics, and biosurfactant production efficiency, strain RM was selected for heavy metal bioremediation studies.

**Fig. 1:**
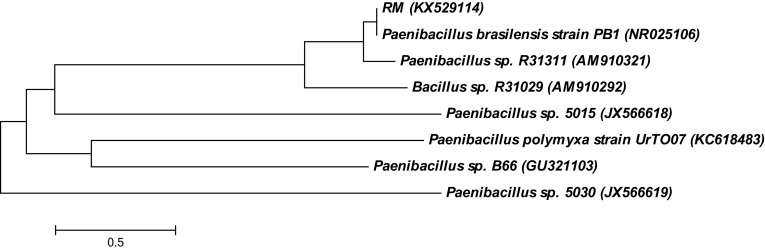
Neighbour-joining tree constructed using Mega 6.0 showing the phylogenetic relationship of 16S rDNA sequence of isolated strain *Paenibacillus* sp. RM from closely related sequences from GenBank. Accession numbers at the GenBank of National Centre for Biotechnology Information (NCBI) are shown in parenthesis

**Fig. 2:**
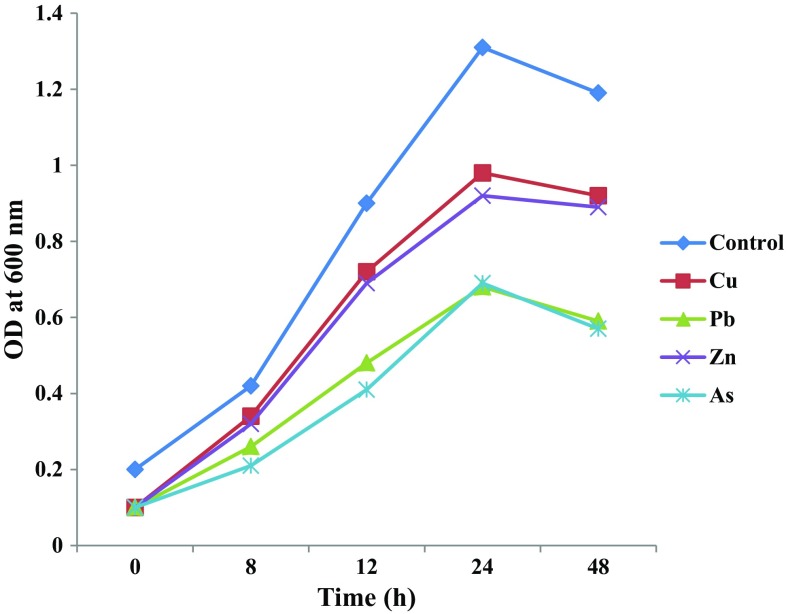
Growth kinetics of *Paenibacillus* sp. RM using various metals (Cu, Pb, Zn, and As) at 100 mg/l

PCR amplification of the 16S rDNA of strain RM resulted in a predicted 1384 bp amplicon. The consensus 16S rDNA sequences of strain RM exhibited 100% similarity with *Paenibacillus brasilensis*. The partial 16S rDNA of strain RM was deposited in GenBank (Accession Number: KX529114). A phylogenetic tree was derived from the partial 16S rDNA sequences of strain RM with existing sequences in the NCBI database, and the results are shown in Fig. 1. The results further confirm the identity of the isolate with *Paenibacillus* sp. Barzanti et al. (2007) isolated metal resistant and plant-growth-promoting endophytic *Paenibacillus* sp. from the roots of Ni-hyperaccumulator *Alyssum bertolonii*. The growth profile of strain RM in the presence of metals is presented in Fig. 2. A minor difference in the growth response was observed according to the metal. The differences in the growth profile could be associated with the toxicity and bioavailability of metals (Govarthanan et al. 2015a).

**Fig. 3:**
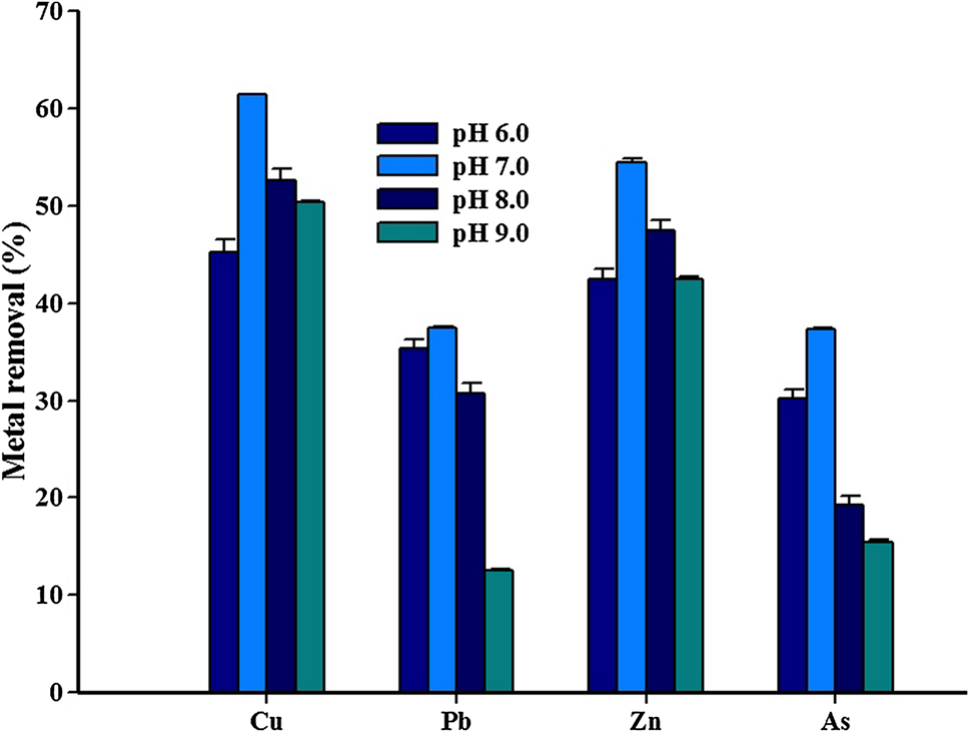
Influence of pH on Cu, Pb, Zn, and As removal by the isolate *Paenibacillus* sp. RM

Cu, Pb, Zn, and As removal efficiency of *Paenibacillus* sp. RM at different pH levels was assessed, and the results are presented in Fig. 3. The removal rates of all the four metals (Cu: 61.4%, As: 37.3%, Zn: 54.5%, and Pb: 37.5%) were high at pH 7.0. A minor decrease in the metal removal rate was observed at pH 6.0. It has been reported that in acidic pH, association of hydronium ions with the cell surface creates reduction of negative charge intensity on the bacterial cell wall resulting in the reduction or inhibition of the binding of metal ions (Khalid et al. 2011). Fourest et al. (1994) reported that the functional groups on the bacteria may become positively charged and may not interact with the metal ions at acidic pH. Similarly, a minor decrease in metal removal capacity was observed with the increase in pH up to 9.0. The decreased removal rate at alkaline pH can be attributed to the growth response of the isolate under pH stress. The limited growth of isolates at alkaline pH reduces the overall uptake of metals (Guo et al. 2010). The results of this study further support the importance of solution pH in metal removal from aqueous solution.

**Fig. 4:**
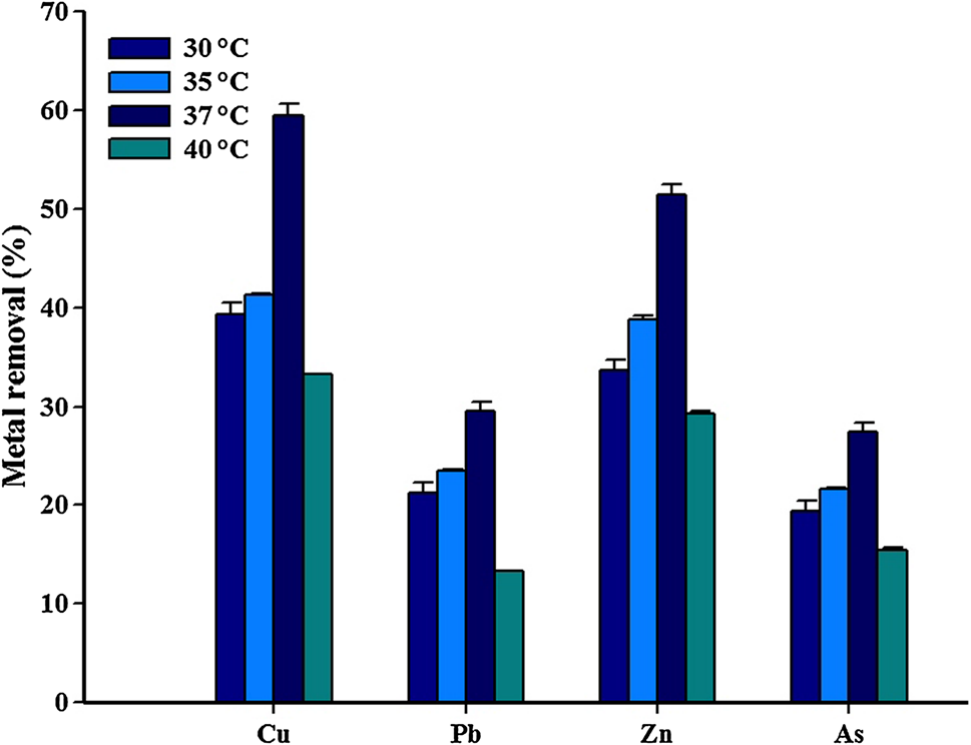
Influence of temperature on Cu, Pb, Zn, and As removal by the isolate *Paenibacillus* sp. RM

**Fig. 5:**
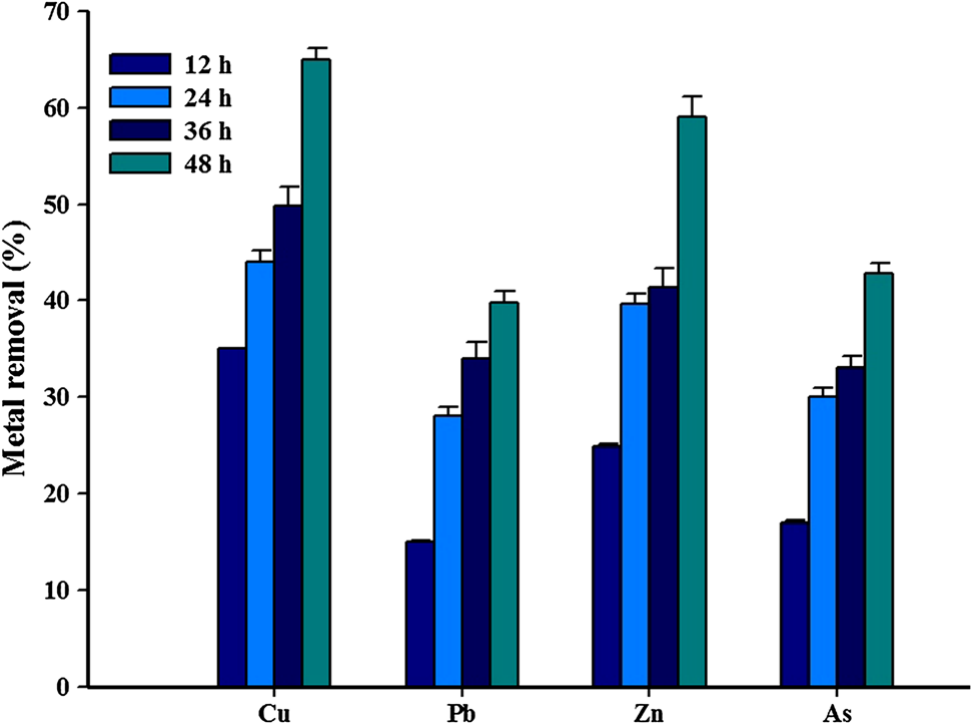
Influence of optimum pH and temperature on Cu, Pb, Zn, and As removal by the isolate *Paenibacillus* sp. RM

Understanding the impact of temperature variations on the metal removal efficiency of bacteria may provide us with valuable insight into the mechanism of interaction between the metal-resistant bacteria and the corresponding metal (Zhou et al. 2013). The metal removal efficiency of *Paenibacillus* sp. was tested with different temperature conditions, as temperature is an important parameter in biological reduction of heavy metals, and the results are presented in Fig. 4. It could be seen from the results of batch experiments that the removal of Cu, Pb, Zn, and As increased with increasing temperature up to 37 °C, but the removal efficiency of all the metal ions decreased slightly at 40 °C. The removal efficiencies of heavy metals at 37 °C were Cu, 59.4%; Pb, 29.5%; Zn, 51.4%; and As, 27.4%. Cu and Zn were significantly removed at 37 °C compared with Pb and As. Zhou et al. (2013) reported the biological removal of Cu and Zn to be highly influenced by temperature. The results from our study indicate that the optimum temperature and pH for the metal removal were 37 °C and 7.0. Thus, the optimum metal removal efficiency of the isolate *Paenibacillus* sp. RM was assessed under optimal conditions, and the results are presented in Fig. 5. Expectedly, all the four metals were removed at higher concentration. The maximum removal rate (65.0%) of Cu was observed at 48 h of incubation, and the minimum removal rate (15.1%) of Pb was observed at 12 h. The bioremediation rate of living metal-resistant bacteria should be strongly dependent on the population of cells at optimal growth conditions (Guo et al. 2010). The results from this study indicated that pH and temperature play a major role in the survival of and metal removal potential of the RM isolate.

## Conclusion

The endophytic bacteria *Paenibacillus* sp. RM isolated from *T. procumbens* showed a significant resistance to Cu, Zn, As, and Pb. Bioremediation studies with batch experiments showed that the isolate *Paenibacillus* sp. RM caused high removal of Cu (59.4%) followed by Zn (51.4). The results indicated the potential role of endophytic bacteria *Paenibacillus* sp. RM in removing multiple metals. Multi-metal resistance and plant-growth-promoting characteristics suggest that the strain *Paenibacillus* sp. RM could be used as a potential candidate for the bioremediation of heavy metals.
